# Supplementary material for: Mn-Containing Bioactive Glass-Ceramics: BMP-2-Mimetic Peptide Covalent Grafting Boosts Human-Osteoblast Proliferation and Mineral Deposition
Source: Materials (Basel). 2022 Jul 1;15(13):4647. doi: 10.3390/ma15134647 (PMC9267458; doi:10.3390/ma15134647)
Supplement: Supplementary file 1 [file materials-15-04647-s001.zip › materials-1763235-supplementary.pdf]

Supplementary materials

Mn-containing bioactive glass-ceramics: BMP-2-mimetic peptide covalent grafting boosts human-osteoblast proliferation and mineral deposition

Leonardo Cassari[1], Paola Brun[2], Michele Di Foggia[3], Paola Taddei[3], Annj Zamuner[1], Antonella Pasquato[1], Adriana De Stefanis[4], Veronica Valentini[4], Vicentiu Saceleanu[5], Julietta V. Rau[6,7,#] and Monica Dettin[1,#,*]

[1]Department of Industrial Engineering, University of Padova, Padova, Italy; leonardo.cassari@phd.unipd.it, annj.zamuner@unipd.it, antonella.pasquato@unipd.it, monica.dettin@unipd.it
[2]Department of Molecular Medicine, University of Padova, Italy; paola.brun.1@unipd.it
[3]Department of Biomedical and Neuromotor Sciences, University of Bologna, Bologna, Italy
[4]Istituto di Struttura della Materia, Consiglio Nazionale delle Ricerche (ISM-CNR), Montelibretti Unit, Via Salaria km 29.300, 00015 Monterotondo Scalo, Italy
[5]Faculty of Medicine, University Lucian Blaga Sibiu, 2A Lucian Blaga Street, 550169 Sibiu, Romania
[6]Istituto di Struttura della Materia, Consiglio Nazionale delle Ricerche (ISM-CNR), Via del Fosso del Cavaliere, 100 - 00133 Rome, Italy
[7]Department of Analytical, Physical and Colloid Chemistry, Sechenov First Moscow State Medical University, Trubetskaya 8, build. 2, 119991 Moscow, Russian Federation
* Correspondence: monica.dettin@unipd.it;
[#] These authors contributed equally to this work

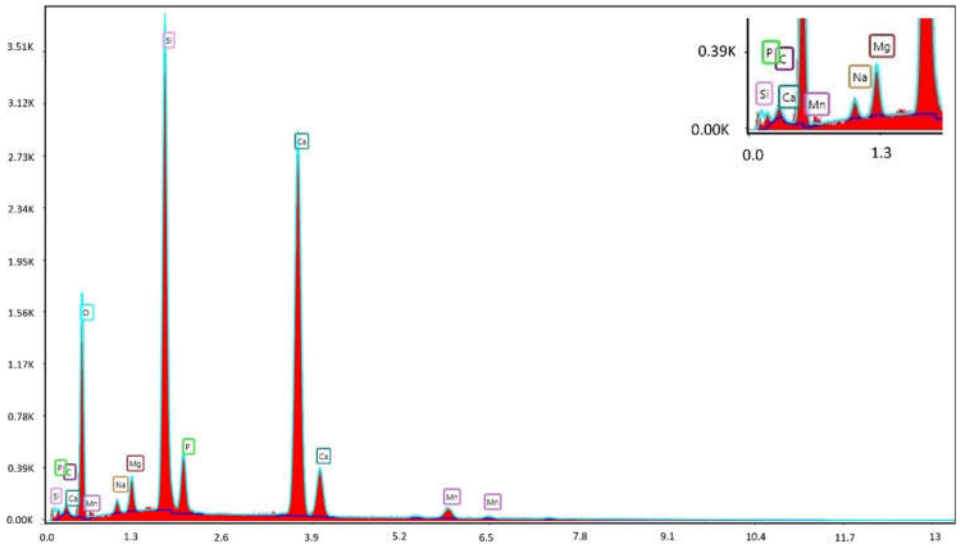

Figure S1. Elemental analysis of the MnGC surface by SEM-EDX. The region of interest is zoomed up in the upper right corner, there is no peak related to nitrogen.
